# Negative Physical Self-Concept Is Associated to Low Cardiorespiratory Fitness, Negative Lifestyle and Poor Mental Health in Chilean Schoolchildren

**DOI:** 10.3390/nu14132771

**Published:** 2022-07-05

**Authors:** Pedro Delgado-Floody, Diego Soto-García, Felipe Caamaño-Navarrete, Bastián Carter-Thuillier, Iris Paola Guzmán-Guzmán

**Affiliations:** 1 Department of Physical Education, Sport and Recreation, Universidad de La Frontera, Temuco 4811230, Chile; pedro.delgado@ufrontera.cl; 2 Department Physical Education and Sports, Faculty of Sport Sciences, University of Granada, 18011 Granada, Spain; 3 Department Physical and Sport Education and Group Research AMRED, University of León, 24007 León, Spain; dsotg@unileon.es; 4 Faculty of Education, Universidad Católica de Temuco, Temuco 4780000, Chile; 5 Departamento de Educación, Programa de Investigación en Deporte, Sociedad y Buen Vivir, Universidad de Los Lagos, Osorno 5290000, Chile; bastiancarter@gmail.com; 6 Faculty of Chemical-Biological Sciences, Universidad Autónoma de Guerrero, Guerrero 39087, Mexico; pao_nkiller@yahoo.com.mx

**Keywords:** mental health, self-concept, fitness, physical activity, schoolchildren

## Abstract

Background: Evidence suggests that physical self-concept (PSC) is linked to well-being in children and adolescents. Objective: The objective was to investigate the association of PSC with mental health (i.e., depression and body image), physical status (i.e., fitness and weight status) and lifestyle (physical activity (PA) patterns and nutritional level) in Chilean schoolchildren. Methods: A total of 617 schoolchildren (*n* = 271 girls and *n* = 346 boys) aged 10–14 years participated in this study. Self-concept, depression and body image dissatisfaction were determined by questionnaires. Physical fitness, PA, screen time (ST), Mediterranean diet (MD) adherence and anthropometric parameters were also included. Results: Poor PSC was linked to bad cardiorespiratory fitness (CRF) (<42 VO_2max_) (OR 1.64; 95%CI 1.12–2.34; *p* = 0.01), severe body image dissatisfaction (OR 2.51, 95%CI 0.99–6.35; *p* = 0.05), ST of more than two hours a day (OR 2.1; 95%CI 1.41–3.12; *p* < 0.001), PA after school of no more than two hours per week (OR 1.52; 95%CI 1.08–2.13; *p* = 0.015) and depression (OR 1.80; 95%CI 1.1–2.92; *p* = 0.017). High nutritional level showed an association with general PSC and general self-concept (*p* < 0.05). Absence of body image dissatisfaction was related to general self-concept (*p* < 0.01) and physical condition dimensions (*p* < 0.05). Conclusions: PSC is associated with CRF, PA after school, ST and nutritional level. According to mental health variables, poor PSC is related to depression in Chilean schoolchildren. Therefore, promoting a healthy lifestyle among children should be a target of community- and school-based interventions to promote PSC.

## 1. Introduction

Childhood and adolescence are crucial periods of life where physical, emotional, psychological and social changes occur [1,2]. In this sense, some studies have pointed out that this age is critical for the development of self-concept, self-knowledge and personal identity [3,4]. Mental health development is a determinant for later life, as mental health is widely seen as an important component of well-being [5]. Good mental health allows an individual to develop their own abilities, to be resistant to the stresses of life, and to make a positive contribution to their peers [6]. Therefore, a low self-concept could reduce children’s potential, increase their susceptibility to stigmatization, and risk their mental and psychological well-being [7].

PSC is defined by self-beliefs about physical ability and perceived physical appearance [8]. Likewise, PSC is configured by the ideas, beliefs or perceptions that are held in the physical field about one’s own ability, strength, attractiveness, physical condition and sports competition, among others [9], usually being defined by culture, age and sex mediators [10]. Self-concept, especially the physical dimension, is very important in the formation of personality. Furthermore, it is related to well-being in general, where by developing a positive self-concept from adolescence, a person can achieve good psychosocial adjustment and avoid future psychological and pedagogical problems [11]. Moreover, a positive self-concept is of the utmost importance for one’s personal, professional and social life, favouring the sense of one’s own identity. Prosocial behaviour also constitutes a frame of reference from which to interpret external reality and one’s own experiences, influences performance, conditions expectations and motivation, and contributes to health and psychological balance [12,13]. Therefore, PSC is fundamental to a wide variety of aspects of human integral development from childhood. For example, studies with K-12 schoolchildren indicate that a positive PSC is associated with the ability to cope with bullying situations and with the development of healthy lifestyle habits such as PA levels [14,15]. Thus, study of the variables and parameters that affect PSC is very important. 

On the other hand, weight status (i.e., according BMI), fitness (i.e., both represent physical status) and lifestyle have been shown to be powerful markers of health [8,16,17] and positive health outcomes including greater well-being and self-concept [8,18]. Moreover, CRF has been associated with optimism and well-being [19]. Similarly, a good lifestyle in terms of PA levels, low ST and good nutritional level is positively associated with well-being [20,21], cognitive performance [22,23] and academic achievement [24]. Some studies have reported a strong association between a low PA level and poor mental and psychosocial well-being [25], and high sedentary time is strongly associated with depressive symptoms [26]. Additionally, it was recently reported in Latin American schoolchildren that negative feelings are associated with unhealthy lifestyles such as low nutritional level such as lack of MD adherence, increased ST, and low PA levels [27]. Likewise, a study of Chilean children showed a positive association between good nutritional level (i.e., MD adherence) and psychological health (i.e., self-esteem and self-concept) [28]. In addition, another study reported a theoretical model suggesting that BMI had a direct effect on PSC, moreover the PA had a positive indirect effect on self-concept in Spanish students [29]. In this sense, the evidence has shown that the self-concept can be negative affected by weight status [30]. Likewise, it has been reported differences in self-concept according fitness levels in schoolchildren [31]. Another study conducted in schoolchildren, showed that children’s self-concept and subjective well-being was related [32]. In addition, a longitudinal study reported that PSC were a fundamental predictor of healthy lifestyle and PSC was linked to health related behaviors an emotions in adolescents girls [33]. A study using a mediation model showed that PSC had a mediating effect in the longitudinal relation between motor ability and well-being in adolescents [34]. Another longitudinal study reported that subjects with more PA levels had better PSC and consequently better well-being [35]. Furthermore, a study conducted on students showed that PSC was related with body image [36]. Likewise, it has been reported that having low PSC impact the risk of having low levels of PA, bad MD adherence and poor life satisfaction in adolescents [37]. Better PA levels and less ST adherence could impact positively the PSC [38,39]

According with the previous evidence, we hypothesized that PSC are linked to well-being, physical fitness and lifestyle at the school age. However, there is little information regarding PSC related to lifestyle parameters that include ST and nutritional level markers such as MD adherence in Chilean children. The objective of the present study was therefore to investigate the association of PSC with psychological well-being (i.e., depression and body image), physical status (i.e., fitness and weight status) and lifestyle (PA, ST and nutritional level) in Chilean schoolchildren.

## 2. Materials and Methods

### 2.1. Participants

This cross-sectional study included 617 schoolchildren (*n* = 271 girls and *n* = 346 boys) aged between 10 and 14 years who attended schools (public and subsidized) from the Araucanía region of Chile. Parents or guardians of all schoolchildren were asked to provide signed consent before participation in this study.

The inclusion criteria were: (i) informed consent from the parents and the assent of the participant; (ii) belonging to an educational centre; and (iii) being aged 10–14 years. The exclusion criteria were: (i) having a musculoskeletal disorder; and (ii) any other known medical condition that might alter the participant’s health and PA levels. Moreover, schoolchildren with physical, sensory or intellectual disabilities were excluded from this study. The research process complied with the Helsinki Declaration (2013) and was approved by the Ethics Committee of Universidad de La Frontera, Chile (ACTAN°086_2017).

### 2.2. Main Outcomes and Independent Variables

#### 2.2.1. Self-Concept (Main Outcomes)

The PSC Questionnaire (CAF) was utilized [40]. A previous study showed that CAF has proved to be adequate to evaluate PSC in Chilean Students [41]. A recent systematic review reported that CAF had high level of validity and reliability in schoolchildren [42]. It is made up of 36 items (20 of them written directly and 16 inversely) that are assessed on a five-point Likert-type scale in which 1 means false and 5 means true. Score ≤120 was reported as low PSC.

PSC is composed of six dimensions:

The physical ability dimension, which students’ express ideas such as “I have no skill in sports” or “I look clumsy in sports activities”. It represents the perception of one’s own ability to practise sport.

The physical condition dimension, which expresses ideas such as “I have a lot of physical energy” or “I can run and exercise for a long time without get tired”. It is related to confidence in one’s own physical state and in the self-perception of stamina to carry out intense physical activities.

The physical appearance dimension, with expressions such as “I find it difficult to have a good physical appearance” or “I feel confident about the physical image I transmit”. This refers to the perception of one’s own physical appearance and the degree of satisfaction one has with the image offered to others.

The strength dimension, which express ideas such as “I am capable of performing activities that require strength” or “I am strong”. It is related to the perception of strength and the ability to carry out activities that require strength, such as lifting weights.

The general PSC dimension, where students express ideas such as “Physically, I am satisfied with myself” or “I feel worse than others”. It represents opinions and feelings (happiness, satisfaction, pride and confidence) in the physical domain.

Finally, the general subscale self-concept, which expresses ideas such as “I feel happy” or “I wish I were different”. This assesses the level at which the subject is satisfied with him/herself and with life in general.

#### 2.2.2. Depression

Characterization of depression symptoms was estimated by the Child Depression Questionnaire (CDI) [43] that consists of 27 groups of three statements each in relation to depressive symptomatology in the last two weeks. For each item, the child has three possible answers: 0, indicating the absence of symptoms; 1, indicating mild symptoms, and 2; indicating definite symptoms. The total score ranges from 0 to 54. There is a suspicion of depression in subjects with values over 18 points. Higher scores indicate higher levels of depression.

#### 2.2.3. Body Image

In terms of body image, the Body Shape Questionnaire (BSQ) [44] was used to identify body image dissatisfaction. The questionnaire was composed of 34 items using a six-point Likert scale, where 1 = never, 2 = rarely, 3 = sometimes, 4 = often, 5 = very often and 6=always. The maximum score that can be obtained is 204 points and a minimum of 34 points. Fewer than 81 points indicates no dissatisfaction with body image; 81–110 points suggests mild dissatisfaction with body image; 111–140 points shows moderate dissatisfaction with body image; and more than 140 points indicates extreme dissatisfaction with body image.

#### 2.2.4. Physical Activity Level

PA levels were measured using the PA Questionnaire (PAQ C) for children. Briefly, the self-administered, seven-day recall questionnaire comprises nine items and collects information on participation in different types of activities and sports (activity checklist); effort during PE; and activity during lunch, after school, in the evening and at the weekend over the past seven days. Each item was scored on a scale of 1–5, the average denoting the PAQ C score [45]. Questionnaire item 10 asks participants whether they were sick last week or whether anything prevented them from doing normal physical activities, results <4 were reported as low general PA.

#### 2.2.5. Lifestyle

The children’s lifestyle was measured according to nutritional level, assessed by the Krece Plus test [46], a tool to determine eating patterns and the relationship with nutritional status based on the MD. The questionnaire has 15 items, and the format assesses a set of items about the food consumed in the diet. Each item has a score of +1 or −1, depending on whether it approximates to the ideal of the MD. The points are added up, and according to the score, the nutritional status is classified as follows: (i) less than or equal to 5 is a low nutritional level; (ii) 6–8 is a moderate nutritional level; and a score greater than or equal to 9 indicates a high nutritional level. This questionnaire has been used in Chilean schoolchildren [21]. The child’s lifestyle was also evaluated by the PA Krece Plus test [46], a quick questionnaire that classifies lifestyle according to the average hours spent watching television or playing video games (ST) daily, and PA hours after school per week. The classification is made according to the number of hours for each item. The total points are added up, and the person is accordingly classified as having either a good lifestyle (male ≥ 9 h, female ≥ 8 h), a regular lifestyle (male 6–8 h, female 5–7 h), or a bad lifestyle (male ≤ 5 h, female ≤ 4 h). The questionnaires were completed individually by the children in the presence of researchers.

#### 2.2.6. Physical Fitness

CRF was estimated by the progressive 20 m shuttle run test (SRT) [47]. The participants were required to run between two lines 20 m apart while keeping pace with audio signals emitted from a pre-recorded CD. The test has been validated among Chilean schoolchildren and has been used in the Physical Education National Study [48]. The results of the 20 mSRT were unified according to the Leger test protocol, and the VO_2max_ was calculated using Leger’s equation [47]: VO_2max_ = (31.025 + 3.238 (V) − 3.248 (A) + 0.1536 (VA)), where V is the velocity in km/h reached in the last stage and A represents the age of the participant [49]. Good values are equal to or higher than 42 ml/kg/min, while low values are less than 42 mL/kg/min according to age and sex [49].

Handgrip muscle strength (HGS) was measured by a hand dynamometer (TKK 5101^TM^, Grip D; Takei, Tokyo, Japan) in order to register upper body strength. The test consists of holding a dynamometer in one hand and squeezing as tightly as possible without allowing the dynamometer to touch the body. Force is applied gradually and continuously for a maximum of 3–5 s [50]. The average of the scores achieved by the left and right hands was registered and used in the analysis.

#### 2.2.7. Anthropometric Parameters

The participant’s body mass (kg) was measured using TANITA scales, model Scale Plus UM–028 (Tokyo, Japan); they were weighed in their underclothes and without shoes. Their height (m) was estimated with a Seca^®^ stadiometer, model 214 (Hamburg, Germany), graded in mm. Body mass index (BMI) was calculated as body mass (kg) divided by the square of the height in metres (kg/m^2^) [51]. Waist circumference (WC) was measured using a Seca^®^ tape measure model 201 (Hamburg, Germany) at the height of the umbilical scar [52]. Waist-to-height ratio (WtHR) was obtained by dividing the WC by height and was used as a tool for estimating the accumulation of fat in the central zone of the body following international standards [53]. In line with recent evidence, a cut-off of ≥0.54 was optimal to considered cardiometabolic risk (CMR) for the Latin American region [54].

#### 2.2.8. Procedure

Research assistants visited the selected school during physical education class day. The data collected were carried out over three separate sessions by a team of researchers trained. Physical fitness was evaluated in the first session. In the second session, anthropometric assessments were carried out in a favorable space facilitated by the school with optimum temperature. Finally, lifestyle surveys and well-being instruments were applied in the classrooms and in the presence of researchers (helped for any potential question).

### 2.3. Statistical Analysis

Statistical analysis was performed using STATA v15.0 software. Normal distribution was tested using the Shapiro–Wilk test. For continuous variables, values are presented at the median, 5th and 95th percentiles. Differences between median values according to sex and risk categories were determined using the Mann–Whitney U test and the Chi-square test, respectively. To determine the relationship between the global self-concept and anthropometric, fitness and lifestyle parameters, a linear correlation was calculated to find the Spearman correlation (rho) coefficient. To determine the association between PSC and individual components, a linear regression was used with the inclusion of beta (95%CI) and odds ratio (95%CI). A radar chart was used to display the multi-dimensional data related to self-concept dimensions, and a comparison between the scores of different associated categories was performed. Values of *p* < 0.05 were considered statistically significant.

## 3. Results

Table 1 shows the participants’ characteristics related to anthropometrics, fitness, lifestyle and self-concept according to sex. There were significant differences only in HGS according to sex (*p* = 0.03). There were no significant differences in the study variables according to prevalence (%) in the other categories (Table 2).

Table 3 shows the relationships and association of global self-concept with anthropometric, fitness and lifestyle parameters in Chilean children. In terms of fitness, VO_2max_ (mL/kg/min) reported a positive association with global self-concept (0.10, 95%CI 0.07–0.13), *p* < 0.001). In relation to lifestyle, ST (h/day) (−0.009, 95%CI; −0.01–−0.004), *p* < 0.001) and nutritional level (−0.03, 95%CI −0.05–−0.01, *p* = 0.002) were inversely linked to global self-concept. Meanwhile, PA after school presented a positive association (0.013, 95%CI 0.007–0.02, *p* < 0.001) with global self-concept. In terms of mental health, depression (−0.04, 95%CI −0.07–−0.01, *p* = 0.002) and body image dissatisfaction (−0.19, 95%CI −0.33–−0.05, *p* = 0.006) were inversely associated with the global self-concept.

Table 4 shows the association of low PSC with anthropometrics, fitness, lifestyle and well-being in Chilean children. Poor PSC was linked to bad CRF (<42 VO_2max_) (OR 1.64, 95%CI 1.12–2.34, *p* = 0.01), severe body image dissatisfaction (OR 2.51, 95%CI 0.99–6.35, *p* = 0.05), ST > 2 h/day (OR 2.1, 95%CI 1.41–3.12, *p* < 0.001), PA after school ≤2 h/week (OR 1.52 95%CI 1.08–2.13, *p* = 0.015) and depression (OR 1.80, 95%CI 1.1–2.92, *p* = 0.017).

**Figure 1:**
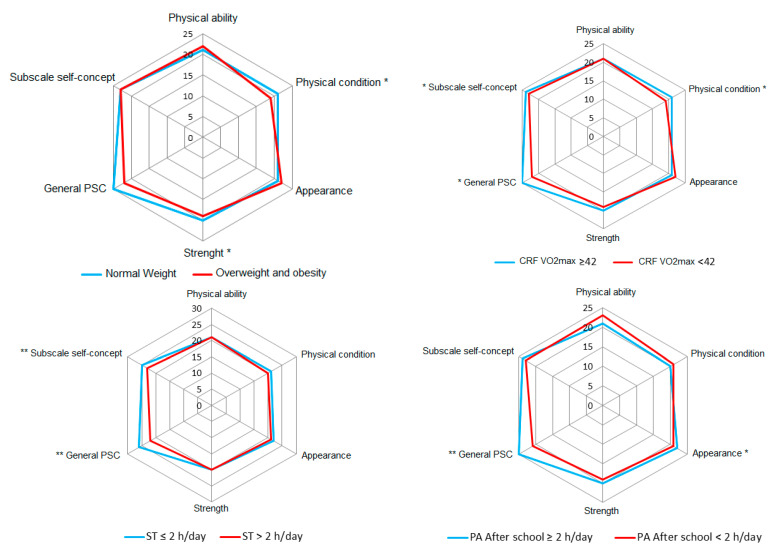

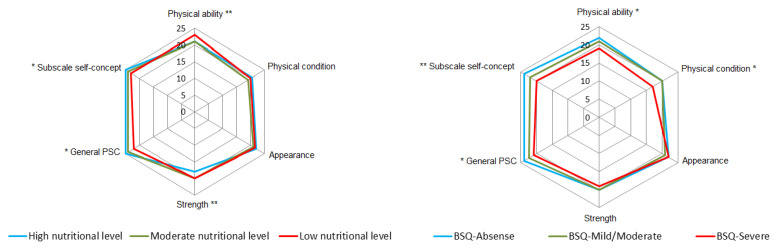
Radar chart shows values of median of different self-concept dimension compared according to anthropometric, fitness, nutritional level and body image categories. The asterisks represent significant statistical differences between categories for each self-concept dimension * *p* < 0.05, ** *p* < 0.01.

Figure 1 charts the median values of the different self-concept dimensions according to anthropometric, fitness, nutritional level and body image categories. In terms of the dimensions of PSC, the analyses showed that normal weight was related positively to strength and physical condition dimensions (*p* < 0.05); good CRF was related positively to global PSC, general self-concept and physical condition (*p* < 0.05); and ST ≤ 2 h/day was linked to global PSC and general self-concept (*p* < 0.01). PA after school ≥2 h/week was linked to global PSC (*p* < 0.01) and appearance (*p* < 0.05). High nutritional level showed an association with general PSC and general self-concept (*p* < 0.05), while low nutritional level was related to physical ability and strength (*p* < 0.01). Finally, absence of body image dissatisfaction was related to general self-concept (*p* < 0.01) and physical condition dimensions (*p* < 0.05).

## 4. Discussion

In the present study, the objective was to investigate the association of self-concept with physical status (i.e., CRF and BMI), PA patterns and psychosocial variables in Chilean schoolchildren. The main findings of this study are, firstly, that depression and body image dissatisfaction are linked to PSC; secondly, that physical fitness and CRF are related to PSC; and thirdly, that lifestyle components are associated with PSC and its dimensions (PA levels were associated with PSC, ST (h/day) was negatively associated with the global self-concept and MD adherence is associated with general PCS and general self-concept).

The evidence have highlighted the importance of PSC of different dimensions such as the well-being [29], healthy lifestyle [33] and body dimensions [55], likewise, the PSC play a fundamental role in the field of psychology in the school age [56]. PSC play a central role in the adolescence due to physical and emotional changes [57]. In relation to psychological well-being variables, the present study reports that poor PSC is linked to depression levels. In this line, it has been indicated that differences in PSC are linked with psychiatric problems [58]. In addition, adolescents with better self-concept and self-esteem had few depression symptoms [59]. Likewise, it has been reported that depression symptoms are related to lower self-concept in schoolchildren [60]. Improve the PSC through PA programs might reduce depression symptoms and increase the subjective well-being at the school [59]. A longitudinal study showed that when the depression score decreased, the self-concept improved in schoolchildren [61]. Likewise, an umbrella systematic review focusing on PA and depression, anxiety and self-esteem in children and young people showed how PA was associated with lower depression and better PSC in children and young people [62].

Likewise, we reported that poor PSC is related to body image dissatisfaction. Similar to our results, another study showed that schoolchildren who had better body image achieved better PSC [2]. In this sense, Sánchez-Miguel et al., indicated that dimensions of self-concept can explained the body dissatisfaction in Spanish adolescents [63]. In addition, a theoretical model indicated that promoting self-concept through PA could positively impact body satisfaction in adolescents [29]. Another study reported a significant relationship between lower self-concept and poorer mental health in adolescents [64]. It is important to note that the evidence reported that the body image dissatisfaction is affected for multiples factors, likewise it is fundamental improve the body satisfaction to reduce future problems related with eating disorders [65].

In our study sample, physical fitness and CRF were related to PSC. This evidence contributes to consolidating the positive association between physical fitness and psychological well-being in schoolchildren [66,67]. In this sense, another study demonstrated that PSC was linked according fitness level in adolescents [31]. Similarly, a previous investigation reported that the components of PSC was associated with physical fitness [68]. Likewise, it has been reported that physical fitness (i.e., CRF, HGS and speed agility) is positively related to high self-concept in schoolchildren [69]. In addition, a previous study showed a positive association between better CRF and psychosocial health factors (i.e., higher self-esteem and body satisfaction, less depression) [70]. Similarly, Reigal et al. [71] reported that CRF was one of the variables of physical fitness that best predicted different dimensions of self-concept. The same authors indicated that students with better physical fitness obtained a better score in psychosocial variables. Another study with 11–17-year-old schoolchildren showed that the relationship between CRF and PA is mediated by PSC, concluding that PSC is an important determinant of PA in adolescents [72]. Current evidence suggests that CRF is a strong predictor of mental health and that improving PA levels will subsequently improve psychological well-being [73].

In the present study, PA levels were associated with PSC. A previous study also reported that PA was positively related to PSC in schoolchildren [38]. Likewise, a systematic review with a meta-analysis reported that PA interventions were linked to higher self-concept in children and adolescents; the authors therefore indicated that PA at school should be increased to promote psychological well-being [74]. In addition, a theoretical model showed that PA mediated by self-concept predicted a better perception of quality of life in schoolchildren [75]. Babic et al. [8] found that PA was positively related to PSC in children and adolescents. In this sense, it was suggested that PA had a positive indirect effect on self-concept in a meditational model in adolescents [29]. Another study that analysed the self-concept before and during the COVID-19 lockdowns showed that PA was linked to better self-concept in adolescents [76]. It is important to note that evidence also shows that high levels of PA are a protective factor against the development and progression of mental health problems [77]. Meeting the PA recommendation during adolescence, ideally at vigorous intensity, can help to prevent and reverse negative mental health situations, being an important educational and therapeutic tool. Likewise, regular practice of PA allows children and adolescents to have a better self-perception and more confidence in themselves [78].

In the present study, ST (h/day) was negatively associated with the global self-concept. Another study that investigated the association of device-measured total sedentary time with self-concept showed that adolescents who engaged in a lot of device-based sedentary time had a lower self-concept [79]. Likewise, it has been indicated that reducing ST could be a promising strategy to promote a better self-concept in adolescents [39]. Furthermore, a longitudinal study indicated that recreational ST was negatively related to mental health [80], while another study indicated that ST was associated with poorer psychosocial well-being over two years in schoolchildren [81]. A study that investigated the association between ST and psychological well-being reported that children who engaged in frequent ST had poorer mental health [82]. Therefore, the evidence has consistently shown a negative association between students’ PSC and sedentary behaviour, while a good PSC was a negative predictor of sitting time [83]. Therefore ST is linked to the development of psychosocial problems in children and adolescents [81]. A longitudinal study indicate that reducing ST can improve the subjective well-being in adolescents [84].

The present study shows that high nutritional level (i.e., MD adherence) is associated with general PSC and general self-concept. A recent study reported that adherence to a MD is linked to subjective well-being in Chilean children [85]. In addition, a recent study conducted among Chilean schoolchildren showed that adherence to a MD was associated with psychological and social health: the authors therefore concluded that it is important to develop healthy food habits in school [27]. Moreover, Grao-Cruces et al. reported that adolescent’s boys with poorer PSC had low MD adherence and poor life satisfaction, therefore the author recommended improve the PSC [37].

### Limitations

The main strength of this study is the examination of several variables that have been implicated in poorer PSC in children. This inclusion generates better understanding of the consequences of physical status, psychological status and lifestyle of children aged 10–14 in Chile. Despite the apparent strengths of this study, the limitations must also be considered. The main limitation of the present investigation is its cross-sectional design. We project in the future longitudinal analyses to clarify the direction of the relations. Another limitation lies in the use of standardized questionnaires to assess lifestyle. This method of assessment is valid to an extent but can allow for the over-estimation of variables when compared to objective device-measured data. Likewise, we used a convenience sample. In the future, we must incorporate intervention studies to see if the variables improve. Improve the lifestyle and physical fitness of students through systematic interventions at the school could be positively impact a better self-concept and subjective well-being.

## 5. Conclusions

In conclusion, PSC is associated with CRF, PA after school, ST and nutritional level. These are all important components of the children’s lifestyle. Mental health variables showed that poor PSC is related to depression in Chilean schoolchildren. Therefore, promoting a healthy lifestyle among children should be a target of community- and school-based interventions to promote PSC.

## Figures and Tables

**Table 1:** Characteristics related to anthropometric, fitness, lifestyle and self-concept in children according to sex.

	Total	Girls	Boys	*p* Value
	*n* = 617	*n* = 271	*n* = 346	
Characteristics				
Demographics				
Age (years)	12 (10–13)	12 (10–13)	12 (10–13)	0.80
Anthropometric parameters				
Body mass (kg)	50.8 (33–79)	51.7 (33–81)	49.9 (33.2–81)	0.17
Zise (m)	1.55 (1.4–1.73)	1.6 (1.4–1.72)	1.55 (1.4–1.73)	0.42
BMI (kg/m^2^)	20.8 (15.6–29.9)	21.4 (15.5–30.7)	20.5 (15.6–29.8)	0.19
WC (cm)	72 (59–97)	72 (59–98)	71 (59–96)	0.15
WHtR (waist/size)	0.46 (0.38–0.60)	0.47 (0.38–0.60)	0.46 (0.38–0.59)	0.26
Fitness				
VO_2max_ (mL/kg/min)	44.5 (35.9–61.8)	44.5 (35.9–59.3)	44.5 (37.7–61.8)	0.46
HGS (kg)	23 (13–42)	22 (12–38)	24 (13–42)	0.03
Lifestyle				
Screen Time (h/day)	3 (1–5)	3 (1–5)	3 (1–5)	0.88
PA afterschool (h/week)	3 (0–5)	3 (0–5)	3 (0–5)	0.12
General PA (score)	2.7 (1.3–5)	2.8 (1.2–5)	2.8 (1.3–5)	0.39
Krece plus (score)	5 (−2–11)	5 (−2–11)	5 (−2–11)	0.57
Mental health				
Body image dissatisfaction (score)	48 (34–134)	47 (34–132)	48 (34–134)	0.40
Depression (score)	13 (4–28)	12 (4–28)	14 (4–28)	0.35
PSC				
Physical ability	21 (13–30)	21 (13–30)	22 (14–30)	0.52
Physical condition	20 (10–30)	20 (10–30)	20 (11–30)	0.90
Appearance	21 (13–30)	22 (13–30)	21 (12–30)	0.33
Strength	20 (10–30)	20 (10–30)	20 (10–29)	0.44
General PSC	23 (11–30)	23 (11–30)	24 (11–30)	0.33
General self-concept	23 (13–30)	23 (13–30)	23 (13–30)	0.99
PSC (total score)	129 (98–160)	129 (98–159)	129 (98–161)	0.93

Data show median and 5th and 95th percentile. BMI = body mass index, WC = waist circumference, WtHR = waist-to-height ratio. VO2max = maximal oxygen consumption. HGS = hand grip strength test. PA = physical activity, BSQ = Body Shape Questionnaire. PSC = physical self-concept. *p* value less than 0.05 are considered statistically significant.

**Table 2:** Characteristics related to anthropometric, cardiorespiratory fitness and lifestyle in children according to sex.

Characteristics	Total *n* = 617	Girls *n* = 271	Boys *n* = 346	*p* Value
Anthropometric parameters				
BMI category				0.25
Normal weight, *n* (%)	311 (50.4)	130 (48.0)	181 (52.3)	
Overweigh, *n* (%)	155 (25.1)	66 (24.3)	89 (25.7)	
Obesity, *n* (%)	151 (24.5)	75 (27.7)	76 (22.0)	
CMR, category, *n* (%)				0.29
WtHR ≤ 0.54	501 (81.2)	215 (79.3)	286 (82.7)	
WtHR > 0.54	116 (18.8)	56 (20.7)	60 (17.3)	
Fitness				
CRF, category				0.36
Good (≥42 mL/kg/min)	415 (67.2)	177 (65.3)	238 (67.3)	
Bad (<42 mL/kg/min)	202 (32.7)	94 (34.7)	108 (31.2)	
HGS, category, *n* (%)				0.40
Acceptable (Up 3er tertile)	423 (68.6)	181 (66.8)	242 (69.9)	
Low (under 3er tertile)	194 (31.4)	90 (33.2)	104 (30.1)	
Lifestyle				
Screen Time, category, *n* (%)				0.86
Acceptable (≤2 h/day)	182 (29.5)	79 (29.1)	103 (29.8)	
Bad (>2 h/day)	435 (70.5)	192 (70.8)	243 (70.2)	
PA afterschool, *n* (%)				0.12
Acceptable (>2 h/week)	361 (58.5)	168 (62.0)	193 (55.8)	
Bad (≤2 h/week)	256 (41.5)	103 (38.0)	153 (44.2)	
PA general, category, *n* (%)				0.87
Good (≥4 score)	143 (23.2)	62 (22.9)	81 (23.4)	
Bad (<4 score)	474 (76.8)	209 (77.1)	265 (76.6)	
Nutritional level, category, *n* (%)				0.97
Hight (≥9)	212 (34.4)	92 (34.0)	120 (34.7)	
Moderate (6–8)	69 (11.2)	31 (11.4)	38 (11.0)	
Low (≤5)	336 (54.4)	148 (54.6)	188 (54.3)	
Body image dissatisfaction, category, *n* (%)				0.64
No	462 (74.8)	205 (75.6)	257 (74.3)	
Mild	85 (13.8)	40 (14.8)	45 (13.0)	
Moderate	51 (8.3)	19 (7.0)	32 (9.2)	
Marked	19 (3.1)	7 (2.6)	12 (3.5)	
Depression, category, *n* (%)				0.65
No	540 (87.5)	239 (88.2)	301 (87.0)	
Yes	77 (12.5)	32 (11.8)	45 (13.0)	

Data shown represent *n* and proportions (%). BMI = body mass index, WC = waist circumference, CMR = cardiometabolic risk, WtHR = waist-to-height ratio, CRF = cardiorespiratory fitness, HGS = hand grip strength test. PA = physical activity. *p*-value less than 0.05 are considered statistically significant.

**Table 3:** Association of physical self-concept with anthropometric, fitness and lifestyle parameters in Chilean children.

Characteristics	Rho Coefficient (*p*-Value)	β (95%CI), *p*-Value
Demographics		
Age (years)	−0.03 (0.32)	−0.002 (−0.006 to 0.002), 0.30
Anthropometric parameters		
Body mass (kg)	−0.05 (0.18)	−0.02 (−0.08 to 0.03), 0.49
BMI (kg/m^2^)	−0.04 (0.24)	−0.007 (−0.02 to 0.01), 0.42
WC (cm)	−0.03 (0.37)	−0.01 (−0.06 to 0.03), 0.51
WHtR (WC/size)	−0.03 (0.44)	−0.00008 (−0.0003 to 0.0002), 0.59
Fitness		
VO2max (mL/kg/min)	0.18 (<0.001)	0.10 (0.07 to 0.13), <0.001
HGS (kg)	0.06 (0.08)	0.02 (−0.01 to 0.05), 0.28
Lifestyle		
Screen Time (h/day)	−0.15 (<0.001)	−0.009 (−0.01 to −0.004), <0.001
PA afterschool (h/week)	0.16 (<0.001)	0.013 (0.007 to 0.02), <0.001
General PA (score)	0.05 (0.17)	0.004 (−0.0008 to 0.008), 0.10
Krece plus (score)	−0.12 (0.001)	−0.03 (−0.05 to −0.01), 0.002
Mental health		
Body image dissatisfaction (score)	−0.11 (0.004)	−0.19 (−0.33 to −0.05), 0.006
Depression (score)	−0.11 (0.003)	−0.04 (−0.07 to −0.01), 0.002

Data shown represent rho Spearman correlation coefficient (*p* value) and β Coefficient (95% CI), adjusted by age and sex. BMI = body mass index, WC = waist circumference, WtHR = waist-to-height ratio, VO2max = maximal oxygen consumption, HGS = hand grip strength test. PA = physical activity. *p*-value less than 0.05 are considered statistically significant.

**Table 4:** Association of low physical self-concept with anthropometric, fitness and lifestyle parameters in Chilean children.

Characteristics	OR (95%CI) *p*-Value
	PSC (≤120 Score)
Anthropometric parameters	
Overweigh or obesity	1.34 (0.96–1.88), 0.08
CMR (WHtR > 0.54)	1.29 (0.84–1.97), 0.22
Fitness	
CRF Bad (<42 Vo_2Max_)	1.48 (1.01–2.16), 0.04
HGS Low (under 3th tertile)	1.03 (0.71–1.47), 0.86
Lifestyle	
Screen Time (>2 h/day)	2.1 (1.41–3.12), <0.001
PA afterschool (≤2 h/day)	1.52 (1.08–2.13), 0.015
General PA (<4 score)	1.50 (0.99–2.28), 0.05
Low Nutritional level (Krece ≤ 5)	0.84 (0.59–1.19), 0.34
Psychological well-being	
Body image dissatisfaction	
Mild or moderate	1.27 (0.85–1.89), 0.24
Severe	2.2 (0.87–5.5), 0.09
Depression	1.80 (1.1–2.92), 0.017

Data shown represent odds ratio (95% CI), adjusted by age and sex. CMR = cardiometabolic risk, WtHR = waist-to-height ratio, CRF = cardiorespiratory fitness, HGS = hand grip strength test. PA = physical activity. *p*-value less than 0.05 are considered statistically significant.

## Data Availability

Not applicable.
